# Baseline-Referenced Longitudinal Point-of-Care Ultrasound for Fluid Management After Kidney Transplantation: A Retrospective Comparison with Central Venous Pressure-Guided Care

**DOI:** 10.3390/life16091567

**Published:** 2026-09-18

**Authors:** Adam Varga, Zoltan Sandor, Dorottya Katics, Karoly Kalmar Nagy, Peter Szakaly

**Affiliations:** Department of Surgery, University of Pecs, 7624 Pecs, Hungary; sandor.zoltan@pte.hu (Z.S.); katics.dorottya@pte.hu (D.K.); kalmar.nagy.karoly@pte.hu (K.K.N.); szakaly.peter@pte.hu (P.S.)

**Keywords:** kidney transplantation, point-of-care ultrasound, POCUS, inferior vena cava, central venous pressure, fluid management, volume status

## Abstract

Fluid management after kidney transplantation requires balancing adequate graft perfusion against fluid overload. We compared central venous pressure (CVP)-guided and point-of-care ultrasound (POCUS)-guided postoperative management and explored whether longitudinal changes in inferior vena cava (IVC) diameter relative to an individual postoperative reference provided information beyond absolute IVC diameter. This single-center retrospective comparative study included 60 adult kidney transplant recipients (30 per group) with standardized assessments on postoperative days 1–5 and 10. POCUS-guided management was associated with more active therapeutic adjustments during days 1–5 (48.0% vs. 25.3%; OR 2.80, 95% CI 1.45–5.42; *p* = 0.002), without a significant difference in cumulative fluid balance. Reference IVC diameter ranged from 7 to 21 mm and showed substantial interindividual variability. Within-patient IVC variation was associated with daily fluid balance (*p* = 0.002) and weight change (*p* = 0.003). In a post hoc exploratory ROC analysis, peak baseline-referenced ΔIVC showed an AUC of 0.749 (95% CI 0.574–0.924) for >1 kg postoperative weight gain, compared with 0.518 (95% CI 0.300–0.737) for peak absolute IVC; the two AUCs were not formally compared. No statistically significant between-group differences in early graft outcomes were observed, although the study was not powered for these outcomes. POCUS-guided monitoring was associated with greater treatment actionability; whether this translates into clinical benefit requires prospective evaluation.

## 1. Introduction

Fluid management during the immediate postoperative period remains a major challenge in kidney transplantation. Adequate circulating volume and arterial pressure are required to support perfusion of the newly transplanted kidney, particularly during the vulnerable period following ischemia–reperfusion. Conversely, excessive fluid administration may contribute to tissue and pulmonary edema, elevated venous pressure, and cardiovascular complications without necessarily improving renal perfusion or graft function [1,2,3]. The clinical challenge is therefore not simply to administer more or less fluid, but to identify the evolving fluid requirements and fluid tolerance of an individual recipient throughout the early postoperative period.

Central venous pressure (CVP) has traditionally been used as a hemodynamic reference during and after kidney transplantation. However, CVP reflects the interaction between right atrial pressure, venous return, right ventricular function, intrathoracic pressure, vascular tone, and other mechanical determinants rather than circulating blood volume alone. Meta-analyses have shown that isolated static CVP values have limited ability to predict fluid responsiveness [4,5]. This does not imply that CVP is physiologically irrelevant. Contemporary interpretation recognizes CVP as a clinically meaningful marker of right-sided filling pressure and systemic venous hemodynamics while emphasizing the limitations of using a single pressure value as a surrogate for intravascular volume or as an isolated trigger for fluid administration [6].

Point-of-care ultrasound (POCUS) offers a complementary approach to bedside hemodynamic assessment. Repeated ultrasonography permits direct visualization of venous and pulmonary physiology and may complement conventional clinical examination. Inferior vena cava (IVC) diameter and respiratory behavior, lung ultrasound, and venous Doppler patterns can provide information regarding right-sided filling conditions, fluid tolerance, and systemic or pulmonary congestion [7,8,9,10]. This multidimensional approach is particularly relevant in nephrology, where body weight and physical examination alone may inadequately characterize the distribution and hemodynamic consequences of extracellular fluid.

Evidence regarding POCUS specifically during the early period after kidney transplantation remains limited but is expanding. Mottola et al. demonstrated that lung ultrasound can identify subclinical postoperative fluid overload following kidney transplantation [11]. Beltramino et al. subsequently reported a substantial prevalence of systemic venous congestion during the first postoperative week in a prospective longitudinal cohort of kidney transplant recipients [12]. More recently, Sosa Barrios et al. showed that an approach incorporating venous excess ultrasound and lung ultrasound detected clinically occult congestion and frequently resulted in modification of postoperative therapy [13]. These studies support the feasibility and potential clinical relevance of postoperative POCUS; however, direct comparisons between POCUS-guided management and conventional CVP-guided care remain scarce.

Interpretation of IVC diameter itself also has important limitations. Absolute IVC dimensions vary considerably among individuals and are influenced by anthropometric and physiological characteristics [14,15]. Respiratory IVC collapsibility in spontaneously breathing patients also has only moderate diagnostic accuracy when used as an isolated marker of fluid responsiveness [16]. Contemporary echocardiographic recommendations therefore interpret IVC size and respiratory behavior primarily within the broader context of right-sided hemodynamics and right atrial pressure estimation rather than as universal measures of intravascular volume [17]. These observations raise the possibility that longitudinal changes relative to an individual patient’s own reference state may contain information that is lost when a population-based diameter threshold is applied.

The primary objective of the present study was to compare routine CVP-guided and POCUS-guided postoperative fluid-management strategies in adult kidney transplant recipients, with particular emphasis on therapeutic adjustment and volume-related outcomes. We hypothesized that POCUS-guided assessment would be associated with more frequent active therapeutic adjustment; cumulative fluid balance and early graft recovery were evaluated as secondary outcomes. As an exploratory objective, we investigated whether baseline-referenced longitudinal changes in IVC diameter provided additional information beyond absolute IVC measurements for characterization of postoperative volume-related changes.

## 2. Materials and Methods

### 2.1. Study Design, Population, and Ethical Approval

This single-center retrospective comparative observational study was conducted at the Department of Surgery, University of Pécs, Pécs, Hungary. Adult kidney transplant recipients undergoing transplantation between 30 January 2024 and 30 May 2025 were considered for retrospective evaluation. During this period, both CVP-guided and POCUS-guided postoperative monitoring were used as components of routine clinical practice. The two monitoring strategies were generally applied in an alternating sequence among consecutive recipients and were not assigned by formal randomization. One exception to the alternating sequence was made for a living-donor–recipient in order to maintain comparable representation of living-donor transplantation between the two monitoring groups, resulting in eight living-donor recipients in each group.

During the study period, 62 consecutive adult kidney transplant recipients underwent transplantation. Of these, 32 were managed using the CVP-guided strategy and 30 using the POCUS-guided strategy. Two recipients from the CVP group were excluded from the retrospective analysis because sufficiently complete and standardized postoperative documentation was unavailable. The final analytical cohort therefore comprised 60 recipients, with 30 patients in each monitoring group. No recipients were retrospectively excluded, exchanged, or substituted on the basis of donor type, graft function, postoperative complications, therapeutic response, or other study outcomes.

The study was conducted in accordance with the Declaration of Helsinki and was approved by the Regional Research Ethics Committee of the University of Pécs Clinical Centre (approval No. 15-PTERKEB2025/2; approval date: 9 February 2026). The present investigation consisted of retrospective analysis of clinical and monitoring data generated during routine patient care. Research-specific retrospective data extraction and statistical analysis were performed after ethics approval had been obtained. The requirement for additional study-specific informed consent was waived. The study is reported in accordance with the Strengthening the Reporting of Observational Studies in Epidemiology (STROBE) statement [18].

### 2.2. Perioperative and Postoperative Management

All recipients underwent kidney transplantation and postoperative treatment according to the same institutional transplant-care principles. Both living- and deceased-donor transplantations were included. Intraoperative fluid-management principles did not systematically differ between the analytical groups; the principal difference concerned the postoperative method used to assess volume status and guide subsequent fluid administration. Isolyte balanced crystalloid solution (Fresenius Kabi Hungary Kft., Budapest, Hungary) was used as the principal maintenance intravenous fluid, and diuretics were not administered routinely. The general postoperative hemodynamic objective was to maintain systolic arterial pressure above approximately 120 mmHg, although no rigid mean arterial pressure-based treatment algorithm was applied. Volume status and fluid therapy were reassessed during the daily morning clinical evaluation.

In the CVP-guided group, central venous pressure was measured through an internal jugular central venous catheter connected to a pressure-transducer system. Measurements were obtained with the patient in the supine position. The transducer was leveled at the phlebostatic axis corresponding to right atrial level and zeroed to atmospheric pressure, and CVP was recorded at end-expiration. A CVP range of 5–10 mmHg served as the principal pressure-based hemodynamic reference. Therapeutic decisions were not based on CVP alone; total output, arterial blood pressure, serum creatinine, body weight, peripheral edema, and the overall clinical condition were considered concurrently.

In the POCUS-guided group, examinations were performed using a Philips InnoSight ultrasound system (Philips Ultrasound, Inc., Bothell, WA, USA) equipped with a C6-2 curved-array transducer. Examinations were performed by three surgeons, each with at least one year of bedside ultrasound experience. All operators followed the same standardized acquisition protocol and anatomical landmarks. Patients were examined lying flat in the supine position. The IVC was visualized from a subcostal/subxiphoid longitudinal window, and maximum end-expiratory diameter was measured perpendicular to the long axis of the vessel approximately 1–3 cm proximal to the IVC-right atrial junction. IVC diameter and respiratory behavior were assessed over three consecutive respiratory cycles during spontaneous breathing, and a representative maximum end-expiratory diameter was recorded. Marked respiratory collapse was considered present when inspiratory reduction in IVC diameter exceeded 50%.

The initial postoperative reference IVC measurement was obtained on the surgical ward after completion of surgery, while the patient was breathing spontaneously and was not receiving vasopressor support. This measurement was used solely as an individual postoperative reference and was not assumed to represent a state of euvolemia. All subsequent postoperative IVC assessments were also performed during spontaneous breathing; none of the included recipients required non-invasive or invasive ventilatory support during these measurements. No universal absolute IVC diameter cutoff was used to determine fluid therapy. Serial IVC measurements were interpreted relative to the recipient’s individual postoperative reference and subsequent trajectory, together with respiratory behavior and the wider clinical context. Lung ultrasound was performed daily. B-lines and pleural effusion were considered sonographic findings suggestive of pulmonary fluid accumulation. IVC distension was interpreted as an increase in diameter relative to the individual reference state accompanied by reduced respiratory collapse rather than according to a predefined diameter threshold. Ultrasound images were not routinely archived as part of clinical practice; therefore, retrospective image-based verification and formal interobserver or intraobserver reproducibility assessment were not possible.

POCUS findings were available to the treating team and were evaluated together with the total output, arterial blood pressure, serum creatinine, body weight, peripheral edema, and the overall clinical picture. Routine graft ultrasonography, including resistive-index assessment, was performed according to standard transplant practice but was not included in the present fluid-management analysis.

Daily therapeutic decisions were derived directly from contemporaneous clinical documentation. The medical record explicitly documented whether fluid administration was increased, maintained using a 1:1 replacement strategy, or restricted, as well as whether dialysis was required. For statistical analysis, these documented treatment instructions were mapped to the corresponding treatment categories. Increased fluid administration primarily consisted of increasing the intravenous infusion rate, whereas fluid restriction consisted of reducing the infusion rate. An active therapeutic adjustment was defined as any decision other than maintenance. The categories were not inferred from subsequent graft function or other downstream study outcomes. Analyses of treatment actionability focused on postoperative days (PODs) 1–5, corresponding to the period of most active early postoperative fluid management.

### 2.3. Data Collection and Outcomes

Clinical data were retrospectively abstracted at the patient level and for PODs 1, 2, 3, 4, 5, and 10. Each recipient therefore contributed six scheduled longitudinal assessments, resulting in 360 postoperative observations. Patient-level data included age, baseline body weight, donor type, day-0 serum creatinine, delayed graft function (DGF), kidney-allograft biopsy, POD10 serum creatinine, and, in POCUS-managed recipients, immediate postoperative reference IVC diameter. Longitudinal data comprised total fluid intake, total output, daily fluid balance, body weight and change from baseline body weight, arterial blood pressure, mean arterial pressure, serum creatinine, lower-extremity peripheral edema, therapeutic decision, and the monitoring variables specific to each strategy.

Total fluid intake incorporated both intravenous and oral intake. Total output incorporated urine output and fluid collected through surgical drains. Daily fluid balance was calculated as total fluid intake minus total measured output. Body weight was measured each morning using the same clinical scale. Peripheral edema was assessed daily by clinical examination, with particular attention to the lower extremities. The core scheduled clinical and monitoring measurements required for the principal analyses were complete.

DGF was defined as the requirement for dialysis during the first seven postoperative days. Kidney-allograft biopsy was performed for graft dysfunction and/or clinical suspicion of acute rejection. Early graft recovery was assessed using serial serum creatinine, POD10 creatinine, percentage creatinine reduction from POD1 to POD5, and time to a reduction of at least 50% from the POD1 creatinine concentration.

Volume-related outcomes included cumulative POD1–5 fluid balance, cumulative positive fluid balance, maximum postoperative weight gain, peripheral edema, and longitudinal patterns of fluid intake and total output. Treatment-process outcomes included the proportion of daily assessments resulting in active therapeutic adjustment or increased fluid administration and the number of such decisions per recipient. Because no single primary endpoint had been prospectively specified before routine clinical data collection, the study was considered exploratory.

### 2.4. Baseline-Referenced IVC Analysis

Baseline-referenced IVC analyses were performed retrospectively and were considered exploratory and hypothesis-generating. Change in IVC diameter was calculated as ΔIVC = IVC_daily − IVC_reference, where IVC_reference was the immediate postoperative measurement obtained on the surgical ward. To separate persistent between-patient differences from longitudinal change within an individual, IVC and CVP measurements were decomposed into recipient-specific mean values and within-person centered values. Each daily measurement was expressed as its deviation from the individual recipient’s serial mean and entered together with the recipient-specific mean into repeated-measures models.

For the patient-level ROC analysis, maximum body-weight change during POD1–5 was used as the clinical outcome. Peak absolute IVC diameter during the same period was compared descriptively with peak change from the postoperative reference IVC, defined as peak POD1–5 IVC minus the immediate postoperative reference IVC. The >1 kg maximum postoperative weight-gain threshold was selected post hoc during exploratory analysis as a pragmatic and readily interpretable categorization of postoperative weight change. It was not prespecified and was not based on a validated transplant-specific cutoff. Accordingly, the ROC analysis was considered exploratory and hypothesis-generating.

### 2.5. Statistical Analysis

Continuous variables are presented as median and interquartile range (IQR), and categorical variables as number and percentage. Between-group continuous variables were compared using the Mann–Whitney U test when appropriate, and categorical variables using Fisher’s exact or chi-square tests according to expected cell counts. Risk ratios with 95% confidence intervals (CIs) were calculated for selected binary patient-level outcomes. Because living-donor representation was intentionally balanced through one deviation from the alternating monitoring sequence, donor type was summarized descriptively and was not subjected to inferential comparison.

Repeated daily observations from the same recipient were not considered statistically independent. Generalized estimating equation (GEE) models with patient as the subject variable and robust standard errors were used for repeated continuous and binary outcomes. Longitudinal models included monitoring strategy, postoperative day, and the monitoring-strategy-by-day interaction. Binary GEE models were used to evaluate active therapeutic adjustment and fluid-increase decisions. Results from binary models are reported as odds ratios (ORs) with 95% CIs.

Recipient-specific mean values and within-person centered monitor values were entered simultaneously into repeated-measures models to separate between-patient from within-patient associations. Intraclass correlation coefficients (ICCs) were estimated from random-intercept models to quantify the proportion of repeated-measure variability attributable to persistent differences between recipients.

Patient-level receiver operating characteristic (ROC) curves were constructed for peak absolute IVC and peak ΔIVC for discrimination of maximum POD1–5 body-weight gain >1 kg. Areas under the curve (AUCs), asymptotic standard errors, and 95% CIs were estimated using nonparametric ROC analysis. Each AUC was tested against the null hypothesis of AUC = 0.5. No formal inferential comparison between the two AUCs was performed; differences in their magnitude were therefore interpreted descriptively.

No formal a priori sample-size calculation was performed. All statistical analyses were performed using IBM SPSS Statistics, version 20 (IBM Corp., Armonk, NY, USA). Tests were two-sided, and *p* < 0.05 was considered statistically significant.

## 3. Results

### 3.1. Study Population and Early Clinical Outcomes

During the study period, 62 consecutive adult kidney transplant recipients underwent transplantation. Thirty-two were managed using the CVP-guided strategy and 30 using the POCUS-guided strategy. Two CVP-managed recipients were excluded because standardized postoperative documentation was incomplete, leaving a final analytical cohort of 60 recipients. Each included recipient contributed six scheduled postoperative assessments, providing 360 longitudinal observations. Baseline characteristics and early clinical outcomes are summarized in Table 1.

Median age was 54.0 years [IQR 43.2–64.8] in the CVP group and 58.5 years [43.0–68.8] in the POCUS group (*p* = 0.416). Median baseline body weight was 74.2 kg [64.0–87.4] and 72.1 kg [60.8–83.6], respectively (*p* = 0.359). Living-donor transplantation accounted for eight recipients (26.7%) in each group following one planned deviation from the otherwise alternating monitoring sequence. Day-0 serum creatinine was 469.5 µmol/L [403.0–685.2] in the CVP group and 568.0 µmol/L [444.2–742.5] in the POCUS group (*p* = 0.395).

Overall postoperative fluid exposure was similar between monitoring strategies. Median cumulative POD1–5 fluid balance was 2580 mL [2047.5–3115.0] in the CVP group and 2430 mL [1912.5–3735.0] in the POCUS group (*p* = 0.853). Cumulative positive fluid balance was likewise comparable at 2580 mL [2077.5–3115.0] and 2590 mL [1965.0–3735.0], respectively (*p* = 0.912), and maximum postoperative weight gain was 0.3 kg [−0.8–1.4] versus 0.5 kg [−0.2–2.0] (*p* = 0.425). Repeated-measures analyses did not demonstrate significant monitoring-strategy-by-day interactions for fluid intake, total output, fluid balance, body-weight change, or serum creatinine.

DGF occurred in 3 of 30 CVP-managed recipients (10.0%) and 2 of 30 POCUS-managed recipients (6.7%; RR 0.67, 95% CI 0.12–3.71; Fisher’s exact *p* = 1.000). Kidney-allograft biopsy was required in two recipients (6.7%) in each group. Median POD10 serum creatinine was 115.0 µmol/L [100.0–152.8] in the CVP group and 119.0 µmol/L [98.8–140.5] in the POCUS group (*p* = 0.530).

Median creatinine reduction between POD1 and POD5 was 59.0% [45.8–72.8] in the CVP group and 66.2% [58.8–71.6] in the POCUS group (*p* = 0.176), while median time to a ≥50% creatinine reduction was 4 days [3.0–5.0] and 3 days [2.0–4.8], respectively (*p* = 0.112). No graft loss, reoperation, intensive-care-unit admission, pulmonary edema, or acute heart-failure event occurred in either group.

### 3.2. Therapeutic Actionability

The most pronounced difference between the monitoring strategies concerned the frequency with which postoperative assessment resulted in modification of fluid therapy. During POD1–5, 38 of 150 CVP-guided assessments (25.3%) resulted in an active therapeutic adjustment compared with 72 of 150 POCUS-guided assessments (48.0%). In GEE analysis accounting for repeated observations within recipients and postoperative day, POCUS-guided management was associated with significantly greater odds of active therapeutic adjustment (OR 2.80, 95% CI 1.45–5.42; *p* = 0.002).

Fluid-increase decisions occurred during 22 of 150 CVP-guided assessments (14.7%) and 62 of 150 POCUS-guided assessments (41.3%), corresponding to an OR of 3.99 (95% CI 1.93–8.25; *p* < 0.001). At the patient level, the median number of active therapeutic adjustments during POD1–5 was 1.0 [0.0–2.0] in the CVP group and 2.0 [1.2–3.8] in the POCUS group (*p* = 0.004). The corresponding numbers of fluid-increase decisions were 0.5 [0.0–1.0] and 2.0 [1.0–3.0], respectively (*p* < 0.001).

Importantly, the higher frequency of therapeutic modification in the POCUS group was not accompanied by greater cumulative fluid exposure (Table 2).

### 3.3. Longitudinal IVC Patterns

**Figure 1:**
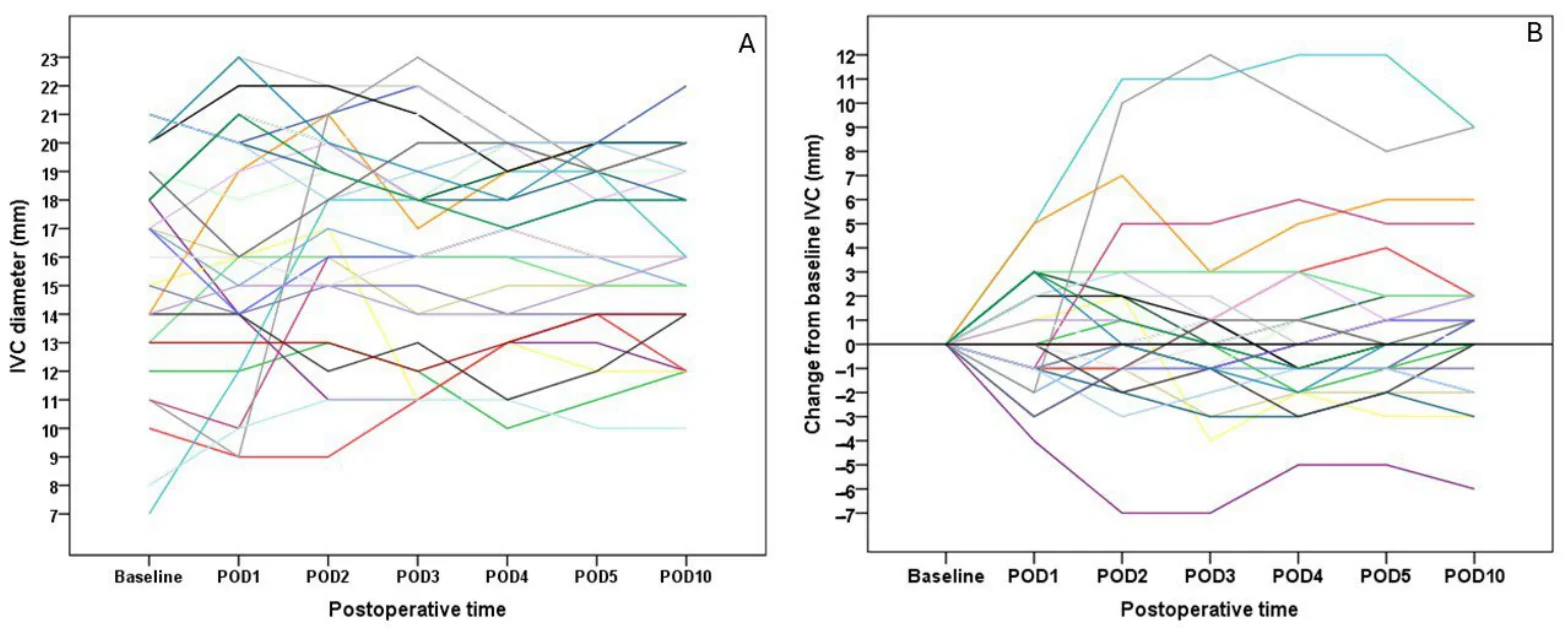
Individual longitudinal inferior vena cava trajectories in POCUS-managed kidney transplant recipients. (**A**) Serial maximum end-expiratory IVC diameters demonstrate substantial between-patient heterogeneity together with relatively consistent individual profiles. (**B**) The same measurements expressed as change from each recipient’s immediate postoperative reference illustrate baseline-referenced longitudinal variation. Reference IVC diameter ranged from 7 to 21 mm, and the intraclass correlation coefficient for serial measurements was 0.794. IVC, inferior vena cava; POD, postoperative day. Different colors are used solely to distinguish individual recipients and do not represent predefined patient groups or categories.

Immediate postoperative reference IVC diameter varied substantially among POCUS-managed recipients, with a median of 17.0 mm [13.2–19.0] and a range of 7–21 mm. Serial measurements demonstrated marked between-patient heterogeneity together with relatively consistent individual trajectories (Figure 1A). When measurements were expressed relative to each recipient’s immediate postoperative reference value, baseline-referenced longitudinal changes were more readily visualized (Figure 1B).

The ICC was 0.794 for serial IVC measurements and 0.637 for serial CVP measurements. Immediate postoperative reference IVC diameter was strongly associated with each recipient’s subsequent mean serial IVC diameter (Spearman ρ = 0.697; *p* < 0.001), whereas reference IVC was not significantly associated with baseline body weight (ρ = 0.240; *p* = 0.202). These findings indicate substantial persistent interindividual differences in serial IVC measurements.

Within-person centered analyses showed that each 1 mm increase in IVC relative to the individual recipient’s characteristic value was associated with a 66.9 mL decrease in concurrent daily fluid balance (β = −66.9 mL/mm; *p* = 0.002) and a 0.195 kg increase in body-weight change (β = 0.195 kg/mm; *p* = 0.003). No significant within-patient association was observed between IVC and total output or serum creatinine.

Within-patient CVP variation was not significantly associated with daily fluid balance (β = −22.4 mL/mmHg; *p* = 0.336), although associations were observed with body-weight change (β = 0.169 kg/mmHg; *p* = 0.031) and serum creatinine (β = −15.0 µmol/L/mmHg; *p* = 0.025).

IVC measurements were also related to therapeutic decisions. Median absolute IVC diameter was approximately 19 mm when the existing fluid strategy was maintained and approximately 16 mm when fluid administration was increased (*p* < 0.001). After within-person centering, each 1 mm increase in IVC was associated with lower odds of a fluid-increase decision (OR 0.476, 95% CI 0.319–0.709; *p* < 0.001). In the CVP group, within-person CVP was not significantly associated with fluid-increase decisions (OR 0.504, 95% CI 0.253–1.006; *p* = 0.052). Following a POCUS-associated fluid-increase decision, next-day IVC diameter increased by a median of approximately 1 mm (*p* < 0.001).

Key longitudinal hemodynamic analyses are summarized in Table 3.

### 3.4. Baseline-Referenced IVC Change and Postoperative Weight Gain

**Figure 2:**
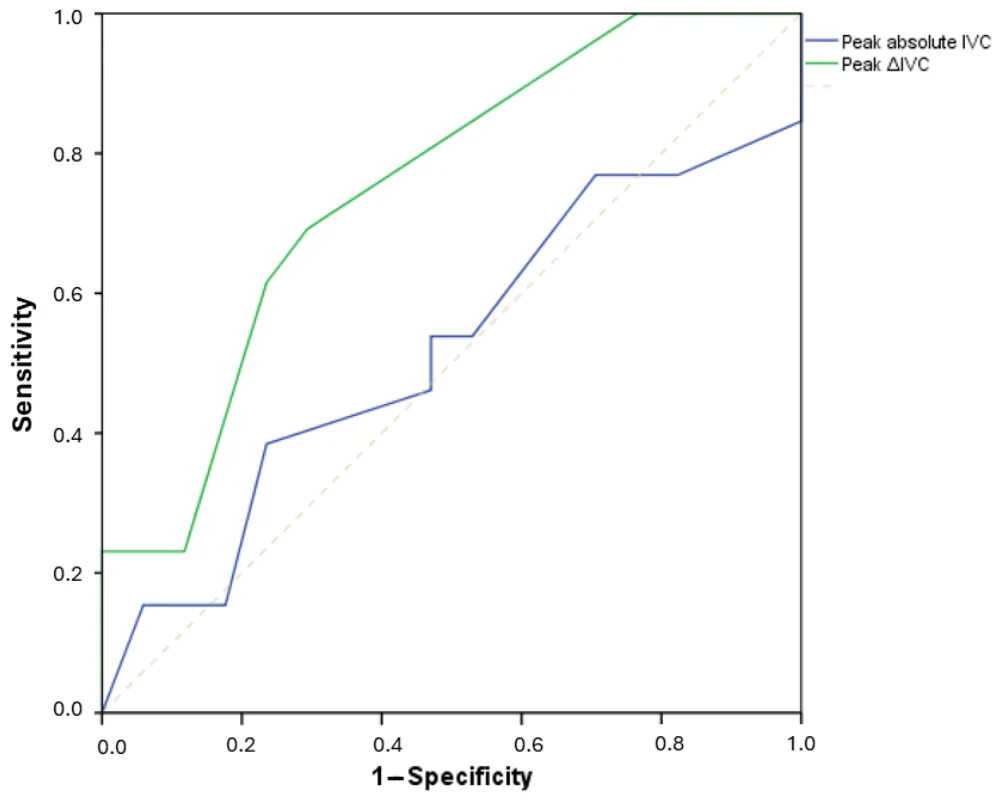
Patient-level receiver operating characteristic analysis of absolute and baseline-referenced IVC measurements for postoperative weight gain. ROC curves compare peak absolute IVC diameter and peak change from the immediate postoperative reference IVC (peak ΔIVC) for the post hoc exploratory outcome of maximum body-weight gain >1 kg during POD1–5 among POCUS-managed recipients. Peak absolute IVC demonstrated an AUC of 0.518 (95% CI 0.300–0.737), whereas peak ΔIVC demonstrated an AUC of 0.749 (95% CI 0.574–0.924; *p* = 0.021 versus an AUC of 0.5). The difference between the AUCs is presented descriptively; no formal inferential comparison between ROC areas was performed. The >1 kg threshold was selected post hoc and should not be interpreted as a validated clinical cutoff. AUC, area under the curve; IVC, inferior vena cava; ROC, receiver operating characteristic. The dashed diagonal line represents the line of no discrimination (AUC = 0.5).

In the post hoc exploratory ROC analysis, 13 of the 30 POCUS-managed recipients experienced a maximum body-weight increase >1 kg during POD1–5. Peak absolute IVC diameter had an AUC of 0.518 (95% CI 0.300–0.737; *p* = 0.867 versus an AUC of 0.5), whereas peak ΔIVC had an AUC of 0.749 (95% CI 0.574–0.924; *p* = 0.021 versus an AUC of 0.5) (Figure 2).

The absolute difference between the observed AUCs was 0.231. Because no formal inferential comparison between the two ROC areas was performed, this difference should be interpreted descriptively rather than as evidence of statistically significant superiority of one marker over the other.

Ultrasound findings interpreted as evidence of fluid overload were documented in four POCUS-managed recipients. Peripheral edema occurred in three of these four patients and in none of the 26 recipients without an ultrasound overload finding (Fisher’s exact *p* < 0.001). Fluid restriction was instituted in three of the four recipients with an ultrasound overload finding and in none of those without such findings (*p* < 0.001). Given the small number of events, formal diagnostic-accuracy estimates were not considered sufficiently stable for emphasis.

## 4. Discussion

In this retrospective comparison of two routinely used postoperative monitoring strategies after kidney transplantation, POCUS-guided management was associated with more frequent active modification of fluid therapy than CVP-guided care. This represents a difference in treatment process rather than evidence of clinical superiority. Cumulative fluid balance did not differ significantly between groups, and no statistically significant between-group differences in early graft outcomes were observed, although the study was not powered for these outcomes. Serial IVC measurements demonstrated substantial interindividual variability, and changes relative to an individual postoperative reference were associated with volume-related variables and therapeutic decisions. The exploratory ROC findings further support prospective study of baseline-referenced longitudinal IVC assessment but do not establish diagnostic or predictive superiority over absolute IVC diameter.

The clearest difference between the monitoring strategies concerned therapeutic actionability. POCUS-guided assessments were associated with nearly threefold greater odds of an active therapeutic adjustment and approximately fourfold greater odds of a fluid-increase decision. More frequent treatment modification should not be equated with improved fluid management or clinical benefit. Additional bedside information may simply have prompted clinicians to modify therapy more frequently, and a higher intervention rate does not establish that these decisions were more accurate, safer, or beneficial. In the present cohort, the higher frequency of treatment modification was not accompanied by a significant difference in cumulative fluid balance. The clinical value of this greater actionability therefore remains unproven and requires prospective evaluation.

Our results add to the limited evidence on POCUS after kidney transplantation. Previous studies have shown that lung ultrasound can identify subclinical postoperative fluid accumulation and that systemic venous congestion may be common during the first postoperative week [11,12]. Sosa Barrios et al. further demonstrated that combined venous and lung ultrasound frequently alters clinical management in the immediate post-transplant period [13]. The present study adds a contemporaneous CVP-guided comparator and quantifies repeated daily therapeutic decisions, illustrating how POCUS can function as a longitudinal monitoring strategy that repeatedly informs treatment decisions.

A particularly relevant observation was the broad interindividual variation in IVC diameter shown in Figure 1. Immediate postoperative values ranged from 7 to 21 mm, while the ICC of approximately 0.79 and the strong association between the postoperative reference and subsequent mean IVC measurements indicated recognizable individual IVC profiles. This finding is consistent with studies demonstrating substantial physiological variation in IVC dimensions and differences in optimal echocardiographic thresholds according to body size [14,15]. It also highlights an important distinction between validated echocardiographic use of the IVC and its application to bedside fluid management. Current recommendations incorporate IVC diameter and respiratory collapse into estimation of right atrial pressure [17]; they do not establish a universal IVC diameter that defines euvolemia or fluid responsiveness across clinical settings.

From a practical perspective, the same absolute IVC diameter may represent different physiological states in different recipients. A given diameter may represent a substantial change relative to one recipient’s postoperative reference while remaining within the typical range of another recipient. This variability provides a physiological rationale for evaluating serial change from an individual reference measurement rather than relying exclusively on a universal absolute threshold. The immediate postoperative reference itself should not be interpreted as a physiological euvolemic baseline. Although it was obtained on the surgical ward during spontaneous breathing and in the absence of vasopressor support, it may still have been influenced by intraoperative fluid administration, anesthesia, and the prevailing perioperative hemodynamic state. Accordingly, ΔIVC represents change relative to an individual postoperative reference rather than deviation from a validated euvolemic state.

The patient-level ROC analysis was consistent with this hypothesis but remains strictly exploratory. Peak absolute IVC diameter had an AUC close to 0.5 for the post hoc >1 kg weight-gain outcome, whereas peak ΔIVC had an AUC of approximately 0.75. Because the two AUCs were not formally compared, these findings do not establish statistical superiority of ΔIVC over absolute IVC diameter. The numerical difference should be interpreted only as a hypothesis-generating observation that warrants prospective testing.

The ROC result should be interpreted cautiously. The >1 kg weight-gain threshold was selected post hoc during exploratory analysis and was not derived from a validated transplant-specific cutoff. Postoperative weight change is itself an imperfect surrogate for intravascular volume and may reflect extracellular fluid redistribution, tissue edema, nutritional intake, and measurement variability. Furthermore, only 30 POCUS-managed recipients contributed to the ROC analysis, and the baseline-referenced measure was developed and evaluated within the same cohort. The present study therefore neither validates a ΔIVC threshold nor establishes ΔIVC as a diagnostic or predictive test for intravascular volume status. The analysis should be regarded as hypothesis-generating evidence supporting prospective evaluation of baseline-referenced longitudinal IVC assessment.

The within-patient analyses showed a similar pattern, although their interpretation is complicated by the observational design. Daily IVC deviations were associated with both fluid balance and body-weight change, whereas within-patient CVP was not significantly associated with daily fluid balance. CVP nevertheless retained associations with body-weight change and serum creatinine in selected models. The patterns of association therefore differed between the two monitoring groups. However, these findings should not be interpreted as a direct physiological comparison between IVC and CVP because the two parameters were obtained in different recipients. CVP remains a direct measure of right atrial pressure and provides clinically relevant information regarding systemic venous hemodynamics; its limitations arise primarily when an isolated value is assumed to represent circulating volume or predict response to further fluid administration [4,5,6]. POCUS, by contrast, can provide visual information from several physiological compartments and may supply additional context unavailable from a single pressure measurement.

A central limitation of the observational design is treatment–measurement feedback. POCUS findings influenced therapy: a relatively larger or less collapsible IVC could prompt reduction in fluid administration, whereas a relatively smaller IVC could trigger fluid escalation. Treatment could in turn alter subsequent fluid balance, body weight, IVC measurements, total output, and serum creatinine. The observed longitudinal associations therefore reflect a dynamic sequence of measurement, clinical interpretation, intervention, and reassessment and cannot be interpreted as untreated causal or independent predictive relationships. They are better viewed as descriptions of monitoring behavior integrated into clinical decision-making, and prospective protocolized studies are required to establish causal effects.

The concept of fluid tolerance is particularly relevant in this context. Conventional postoperative management has traditionally emphasized adequate preload and arterial pressure, whereas systemic and pulmonary venous congestion may coexist with apparent preload dependence [8,9,10]. In kidney transplant recipients, systemic venous congestion may theoretically impair the pressure gradient supporting graft perfusion and contribute to renal interstitial congestion. Although ultrasound overload findings were uncommon in the present cohort, their association with peripheral edema and fluid restriction was physiologically coherent. The small number of events precludes reliable diagnostic-performance estimates. Our focused protocol incorporated IVC and lung ultrasound but did not constitute a comprehensive multiorgan venous-congestion assessment. More comprehensive venous Doppler approaches, including the hepatic, portal, and intrarenal components of the VExUS framework, represent a logical direction for future transplant-specific studies.

No statistically significant between-group differences were observed in DGF, biopsy requirement, POD10 serum creatinine, early creatinine reduction, or time to 50% creatinine decline. However, only five DGF events occurred, and the study was not powered to detect clinically meaningful differences in these or other infrequent outcomes. The absence of statistically significant differences therefore should not be interpreted as evidence of equivalence, non-inferiority, or safety, nor as evidence that POCUS improves graft recovery. Determining whether individualized POCUS-guided management can reduce DGF, shorten hospitalization, or improve longer-term graft function requires a substantially larger prospective study.

The present findings suggest a potential framework for future postoperative POCUS protocols after kidney transplantation. Rather than reducing volume assessment to a single target value, such protocols could integrate the recipient’s longitudinal filling trajectory with markers of fluid tolerance, including respiratory IVC behavior and pulmonary or systemic venous congestion, together with clinical response variables such as total measured output, serum creatinine trajectory, body-weight change, arterial pressure, and standard graft ultrasonography. Prospective studies should determine whether baseline-referenced changes add clinically useful information beyond conventional assessment and whether more comprehensive multiorgan congestion frameworks improve postoperative decision-making.

Several limitations should be acknowledged. First, this was a single-center, non-randomized observational study. Although the two monitoring strategies were generally applied in an alternating sequence among consecutive recipients rather than selected according to clinical status, formal randomization was not performed and residual confounding cannot be excluded. One deviation from the alternating sequence was made to maintain comparable representation of living-donor transplantation between groups. Because the two strategies were interspersed throughout the same study period, major period effects are less likely than in a before–after design, although temporal changes in perioperative practice cannot be fully excluded. Of 62 consecutive recipients, two CVP-managed patients were excluded because standardized postoperative documentation was incomplete; although no exclusions were based on clinical outcomes, some risk of selection bias remains. Not all potentially relevant perioperative and transplant-specific covariates were available for consistent retrospective analysis, which may contribute further residual confounding. Second, no formal a priori sample-size calculation or prospectively defined primary endpoint was available, and the study was underpowered for infrequent clinical outcomes such as DGF or other major postoperative complications. Nonsignificant between-group differences therefore cannot establish equivalence or safety. Third, treatment–measurement feedback is intrinsic to the study design because monitoring findings influenced treatment and treatment subsequently influenced the physiological variables being measured, limiting causal and predictive interpretation of longitudinal associations. Fourth, POCUS examinations were performed by three operators with at least one year of ultrasound experience using a standardized protocol, but formal interobserver and intraobserver reproducibility testing was not performed. Images were not routinely archived, precluding retrospective blinded verification. These factors may limit reproducibility and generalizability to centers with different levels of POCUS expertise. The focused protocol incorporated IVC and lung ultrasound but not a comprehensive multiorgan venous-congestion assessment such as VExUS. Finally, body-weight change, peripheral edema, and IVC behavior are surrogate measures and do not constitute independent gold-standard assessments of intravascular volume. The >1 kg weight-gain threshold was selected post hoc, and the ROC analysis involved only 30 POCUS-managed recipients and no formal comparison of ROC areas. These findings should therefore be regarded as exploratory and hypothesis-generating and require prospective external validation.

Despite these limitations, the study has several strengths. It represents a near-complete consecutive single-center cohort, with 60 of 62 recipients included, and directly compares two clinically relevant monitoring strategies used under the same general perioperative treatment principles. Six standardized postoperative assessments were available for each included recipient, and the statistical analyses accounted for repeated observations within patients. The availability of an immediate postoperative reference IVC enabled baseline-referenced longitudinal analyses, while the patient-level ROC approach avoided treating serial observations as independent. Importantly, the study assessed not only physiological associations but also whether monitoring information translated into actual changes in bedside treatment.

## 5. Conclusions

POCUS-guided postoperative monitoring after kidney transplantation was associated with more frequent active adjustment of fluid therapy than CVP-guided care, while cumulative fluid balance did not differ significantly between groups. No statistically significant differences in early graft outcomes were observed, although the study was underpowered for these outcomes. Longitudinal IVC measurements showed substantial interindividual variability, and changes relative to an individual postoperative reference were associated with fluid-related variables and therapeutic decisions. Exploratory analysis suggested that baseline-referenced IVC change may warrant further investigation as a longitudinal monitoring measure, but the present study does not establish diagnostic superiority or clinical benefit.

These findings support prospective evaluation of baseline-referenced longitudinal POCUS approaches rather than reliance on universal IVC thresholds or isolated static pressure measurements. Larger, adequately powered studies using predefined individualized POCUS algorithms and clinically meaningful endpoints are required to determine whether this strategy improves graft or patient outcomes.

## Figures and Tables

**Table 1:** Baseline characteristics and early clinical outcomes. Values are median [IQR] or n (%). Living-donor representation was intentionally balanced through one deviation from the alternating monitoring sequence; therefore, no inferential comparison is presented for donor type. IQR, interquartile range; POD, postoperative day.

Variable	CVP-Guided (*n* = 30)	POCUS-Guided (*n* = 30)	Effect/ *p* Value
Age, years	54.0 [43.2–64.8]	58.5 [43.0–68.8]	0.416
Baseline body weight, kg	74.2 [64.0–87.4]	72.1 [60.8–83.6]	0.359
Living-donor transplantation	8 (26.7%)	8 (26.7%)	
Day-0 creatinine, µmol/L	469.5 [403.0–685.2]	568.0 [444.2–742.5]	0.395
Delayed graft function	3 (10.0%)	2 (6.7%)	RR 0.67 (95% CI 0.12–3.71); *p* = 1.000
Kidney-allograft biopsy	2 (6.7%)	2 (6.7%)	1.000
Peripheral edema during POD1–5	6 (20.0%)	3 (10.0%)	0.472
POD10 creatinine, µmol/L	115.0 [100.0–152.8]	119.0 [98.8–140.5]	0.530

**Table 2:** Therapeutic actionability and selected volume-related outcomes during POD1–5. (Values are n/N (%) or median [IQR]. OR, odds ratio; CI, confidence interval; POD, postoperative day.)

Outcome	CVP-Guided	POCUS-Guided	Effect/*p* Value
Active therapeutic adjustments	38/150 (25.3%)	72/150 (48.0%)	OR 2.80 (95% CI 1.45–5.42); *p* = 0.002
Fluid-increase decisions	22/150 (14.7%)	62/150 (41.3%)	OR 3.99 (95% CI 1.93–8.25); *p* < 0.001
Active adjustments per patient	1.0 [0.0–2.0]	2.0 [1.2–3.8]	*p* = 0.004
Fluid increases per patient	0.5 [0.0–1.0]	2.0 [1.0–3.0]	*p* < 0.001
Cumulative fluid balance, mL	2580 [2047.5–3115.0]	2430 [1912.5–3735.0]	*p* = 0.853
Maximum weight gain, kg	0.3 [−0.8–1.4]	0.5 [−0.2–2.0]	*p* = 0.425

**Table 3:** Longitudinal hemodynamic analyses. (IVC, inferior vena cava; CVP, central venous pressure; OR, odds ratio; CI, confidence interval.)

Analysis	Estimate	*p* Value
Postoperative reference IVC diameter	17.0 [13.2–19.0] mm; range 7–21 mm	-
IVC intraclass correlation coefficient	0.794	-
CVP intraclass correlation coefficient	0.637	-
Reference IVC vs. serial mean IVC	ρ = 0.697	<0.001
Within-patient IVC vs. daily fluid balance	β = −66.9 mL/mm	0.002
Within-patient IVC vs. weight change	β = 0.195 kg/mm	0.003
Within-patient CVP vs. daily fluid balance	β = −22.4 mL/mmHg	0.336
Within-patient CVP vs. weight change	β = 0.169 kg/mmHg	0.031
Within-patient IVC vs. fluid-increase decision	OR 0.476 (95% CI 0.319–0.709)	<0.001
Within-patient CVP vs. fluid-increase decision	OR 0.504 (95% CI 0.253–1.006)	0.052

## Data Availability

The data supporting the findings of this study are available from the corresponding author upon reasonable request. Public availability of individual-level clinical data is restricted by institutional and ethical requirements regarding patient confidentiality.
